# Harnessing Rhizobial Inoculation for Sustainable Nitrogen Management in Mung Bean (*Vigna radiata* L.)

**DOI:** 10.3390/plants14233695

**Published:** 2025-12-04

**Authors:** Dieini Melissa Teles dos Santos, Vinício Oliosi Favero, Ana Beatriz Carneiro Leite, Giulia da Costa Rodrigues dos Santos, Jaqueline Carvalho de Almeida, Josimar Nogueira Batista, Willian Pereira, Everaldo Zonta, Segundo Urquiaga, Norma Gouvêa Rumjanek, Gustavo Ribeiro Xavier

**Affiliations:** 1 Department of Agronomy, Federal Institute of Education, Science, and Technology of Rondônia, Campus Colorado do Oeste, Vilhena 76993-000, RO, Brazil; 2 Department of Soil, Institute of Agronomy, Federal Rural University of Rio de Janeiro, Seropédica 23897-000, RJ, Brazil; vinicio.favero@incaper.es.gov.br (V.O.F.); ezonta@ufrrj.br (E.Z.); 3 Capixaba Institute for Research, Technical Assistance and Rural Extension (INCAPER), São Gabriel da Palha 29780-000, ES, Brazil; 4 Institute of Agronomy, Federal Rural University of Rio de Janeiro, Seropédica 23897-000, RJ, Brazil; beatriz-leite@hotmail.com.br (A.B.C.L.); giuliasantos@ufrrj.br (G.d.C.R.d.S.); 5 Biological Resource Center, CRB, Embrapa Agrobiology, Seropédica 23897-970, RJ, Brazil; jaqueline.carvalho@colaborador.embrapa.br; 6 Campus Campos dos Goytacazes, Federal Rural University of Rio de Janeiro, Campos dos Goytacazes 28022-560, RJ, Brazil; josimarbatista@ufrrj.br (J.N.B.); willianpereira@ufrrj.br (W.P.); 7 Department of Plant Science, Institute of Agronomy, Federal Rural University of Rio de Janeiro, Seropédica 23897-000, RJ, Brazil; 8 Embrapa Agrobiology, Seropédica 23897-970, RJ, Brazil; segundo.urquiaga@embrapa.br (S.U.); gustavo.xavier@embrapa.br (G.R.X.)

**Keywords:** biological nitrogen fixation, bioproducts, plant growth promotion, pulse crop sustainable grain production

## Abstract

As a pulse crop, mung beans are associated with nitrogen-fixing bacteria, which can improve soil fertility, lower the need for nitrogen fertilizers, and increase yield and soil quality for subsequent harvests. This study aimed to identify effective rhizobial inoculants for mung beans (*Vigna radiata* L.) by evaluating selected strains for cowpea (*Vigna unguiculata* L.), soybean (*Glycine max* L.), and common bean (*Phaseolus vulgaris* L.) under controlled (axenic) conditions. Cowpea, soybean, and common bean strains were tested as mung beans inoculants under axenic conditions. Promising strains were then tested in the field to assess grain yield and to quantify nitrogen fixation using the ^15^N natural abundance method. The cowpea strain BR 3302 (*Bradyrhizobium viridifuturi*) increased mung bean yield by 18%, achieving results similar to a 240 kg N ha^−1^ fertilizer application. The soybean strain BR 96 (*B. elkanii*) facilitated the highest nitrogen fixation (35.3 kg N ha^−1^), significantly surpassing the contribution of indigenous diazotrophic bacteria (18.5 kg N ha^−1^). Interestingly, BR 3302 appeared to primarily enhance nitrogen uptake from the soil (65% of plant N), indicating the presence of other potential plant growth-promoting mechanisms beyond nitrogen fixation. This research demonstrates that *Bradyrhizobium* strains can benefit mung beans through both enhanced nitrogen fixation and additional growth-promoting mechanisms, offering a sustainable approach to improve mung beans production.

## 1. Introduction

Pulses comprise a group of crops consumed as dry beans, rich in protein and fiber, that play an essential role in human nutrition and thus represent a valuable global resource for food security [1]. Most pulses are well-suited to stress. In part, this ability to thrive in low-fertility soils is given by biological nitrogen fixation (BNF) carried out by rhizobia that inhabit the root nodules of these plants, as well as by pulse plant growth promoting rhizobacteria (PGPR) [2].

Native to the India-Burma region, mung beans (*Vigna radiata* L.) are widely grown across Asia, making up nearly 90% of the world’s production [3]. While India is the top producer, with an average annual output of 1.48 million tons, its grain yield is still low at about 470 kg per hectare [1].

In Brazil, mung beans have become popular among medium- and large-scale farmers due to their high productivity (averaging 2000 kg ha^−1^), resistance to water stress, and ability to withstand high temperatures [4]. In 2021, mung beans were the pulse with the highest export volume, generating over 67.6 million dollars in revenues for Brazil. They have significant potential to expand cultivation areas [5] and face high export demand in Asian countries.

The predominant mung bean symbiont in Brazilian agriculture is *Bradyrhizobium*. Among the species, *B. yuanmingense* shows the most significant increase in shoot biomass [6]. This genus significantly improves nodulation, root, and shoot biomass, while strains from the B. japonicum superclade strains perform better than those from the *B. elkanii* superclade [7]. *Bradyrhizobium* also nodulates mung beans in Ethiopia, a hotspot for rhizobial diversity [8]. Other rhizobia (*Rhizobium*, *Mesorhizobium*, *Ensifer*) and non-rhizobial endophytes (*Leifsonia*, *Bacillus*, *Agrobacterium*) have been isolated from mung bean nodules [6]. Fast-growing species such as *E. aridi*, *E. meliloti*, and *R. pusense* can also form effective associations with mung beans [9].

Research shows that mung bean nodulation with native rhizobia is generally lower in the Brazilian Cerrado (tropical savanna) compared to the Atlantic Forest [6]. Without a specific inoculant, producers have used inoculants recommended for other crops to enhance BNF. Therefore, studies evaluating the performance of strains already registered for other legumes can speed up the availability of inoculation technology for mung beans. In this context, it is important to assess how cross-inoculation affects grain yield under specific soil and climate conditions. The objective of this study is to select inoculants for mung beans from commercial strains already recommended for cowpea, common bean, and soybean.

## 2. Materials and Methods

### 2.1. Selection of Rhizobial Strains for Mung Bean Under Controlled Conditions

An assay was conducted in a greenhouse under axenic conditions at Embrapa Agrobiologia, in the municipality of Seropédica-RJ, using Leonard jars, with two compartments [10]. A randomized block experimental design was used with 12 treatments (11 strains and a non-inoculated control) and five replicates, in a total of 60 jars.

The following strains registered at MAPA (Normative Instruction No 13, 25 March 2011; MAPA, 2013) were evaluated: four from cowpea-SEMIA 6463, SEMIA 6461, SEMIA 6462, and SEMIA 6464; four from soybean-SEMIA 5080, SEMIA 5079, SEMIA 587, and SEMIA 5019; and three from common bean-SEMIA 4077, SEMIA 4088, and SEMIA 4080 (Table 1). All strains were provided by the Johanna Döbereiner Biological Resources Center, Embrapa Agrobiologia, Seropédica, Brazil, registered with the World Federation for Culture Collections (WFCC, 364).

The Camaleão mung bean cultivar (MAPA registry 36829) was developed by the Minas Gerais Agricultural Research Agency, Belo Horizonte, Brazil (asagro@epamig.br) and released in 2018. Seeds were disinfected with 70% alcohol (for 60 s), hydrogen peroxide (for 3 min), and washed several times in autoclaved distilled water. Five seeds were used per jar.

Rhizobial isolates were inoculated into YM liquid culture medium (containing yeast extract and mannitol) and incubated on an orbital shaker (New Brunswick Scientific Company, Inc., Edson, NJ, USA) at 30 °C and 150 rpm [18]. Strains were grown for three to seven days, reaching a concentration of 10^8^ CFU mL^−1^. After growth, 1 mL of the bacterial suspension, which results in an approximate concentration of 10^8^ CFU per seedling, was placed at the collar of each seedling using an automatic pipette. Five days after emergence (DAE), one plant was left per jar.

Norris’s solution (N-free), prepared according to the instructions in the Supplementary S2, was added weekly by adding 300 mL to the lower compartment of the Leonard jar [10,19]. The upper compartment was filled with a substrate made of sterilized gravel and vermiculite (2:1 *v*/*v*), and seeds were then sown.

Plants were collected at 60 DAE. The shoot was cut at the height of the cotyledon node, the roots were washed, and the nodules were detached and counted. Shoot, root, and nodule biomasses were dried in a forced-air oven at 60 °C until reaching constant mass. Nodule number and the dry masses of nodule, root, and shoot were evaluated.

### 2.2. Field Evaluation of Bradyrhizobium Strains as Inoculants for Mung Bean

The assay was conducted from February to April 2020 in the experimental area of the Federal Rural University of Rio de Janeiro, located in the municipality of Campos dos Goytacazes, RJ (21°48′10.81” S, 41°17′43.14” W), at an altitude of 10 m above sea level in the Southeast region of Brazil within the Atlantic Forest Biome. The region’s climate is classified as tropical (Aw) according to the Köppen–Geiger classification [20], characterized by dry winters and hot summers with rainfall occurring from October to March. Total precipitation during the experiment was 363 mm, and the average maximum and minimum temperatures were 30 and 21 °C, respectively. The soil is a Haplic Cambisol [21] and its chemical properties determined from a composite sample collected at a depth of 0–20 cm, were: 28.1 g dm^−3^ of organic matter, 27 mg dm^−3^ of P, 105 mg dm^−3^ of K^+^, 897.7 mg dm^−3^ of Ca^2+^, 340.3 mg dm^−3^ of Mg^2+^, and a pH in water of 5.9.

The experiment was conducted in a randomized block design with 6 treatments and 4 replications, yielding 24 plots. Each plot measured 4.0 m wide and 6.0 m long, with 1.0 m spacing between plots, and eight rows spaced 0.50 m apart. The treatments included four strains selected from the greenhouse experiment: three from cowpea SEMIA 6461 (=BR 3302) of *Bradyrhizobium viridifuturi* [12], SEMIA 6463 (=BR 3301) of *B. amazonense* [11], and SEMIA 6462 (=BR 3267) of *B. yuanmingense* [13]; one from soybean SEMIA 587 (=BR 96) of *B. elkanii* [14,15]; and two controls: a nitrogen control receiving 240 kg N ha^−1^ (without inoculation) and a non-inoculated control (without inoculation and nitrogen fertilization).

The inoculants were prepared under controlled laboratory conditions to ensure cell viability and uniformity, following the Standard Operating Procedure titled “Cell Culture for the Production of Inoculants,” Embrapa Agrobiologia: Seropédica, Brazil, 2004 [22] Each bacterial strain was initially grown in test tubes with 5 mL of YM medium [18], supplemented with 500 μL of a stock suspension stored at −80 °C. The cultures were incubated on a rotary shaker at 150 rpm and 30 °C for 24 h. Subsequently, the entire content of each tube was transferred to a 500 mL Erlenmeyer flask containing 50 mL of YM medium and incubated again under the same conditions for an additional 24 h. After incubation, 25 mL of the culture was aseptically mixed with 40 g of autoclaved, sterilized peat that had been previously autoclaved at 121 °C for 30 min and stored in sealed containers. The cell concentration of the inoculant was approximately 10^8^ CFU mL^−1^.

Fertilizer was applied along the sowing row at planting: 50 kg P_2_O_5_ per hectare, 50 kg K_2_O per hectare, and 240 kg N per hectare. The nutrients were divided: one-third applied at 7 DAE and two-thirds at the start of flowering (23 DAE) on the surface. The soil’s pH did not require adjustment. Only the nitrogen control plots received urea fertilizer.

Seeds from a local mung bean variety, known as “Duque,” have been selected after several years of continuous cultivation since its introduction at Embrapa Agrobiologia in the 1980s. Seeds are available at the Integrated Agroecological Production System-“Fazendinha Km 47,” located at Rua B, 1048, Ecologia, Seropédica-RJ (Rodovia BR 465, CEP 23890-000, telephone +55-(21)-2682-1082).

Seeds were pre-inoculated with 10^4^ CFU per seed, and planting was performed manually with 15 seeds per linear meter, resulting in a population density of 300,000 plants per hectare. Sprinkler irrigation was applied every three days during a drought.

The variables analyzed at 23 DAE included nodule number and the dry masses of nodules, roots, and shoots. Five plants were collected from each plot at the height of the cotyledonary node, in the second row from the edge, 1.0 m from the boundary. Roots were washed, and nodules were detached, counted, and dried in a forced-circulation oven at 60 °C along with roots and shoots until reaching a constant mass. At 44 DAE, between flowering and the start of grain filling, the following variables were evaluated: shoot dry mass, N content, total N accumulated in the shoot, N content derived from air (Ndfa), accumulated N derived from BNF (N-fixed), accumulated N derived from soil (soil N-uptake), and the partition of shoot N derived from air and soil. Five plants were collected from each plot in the second row, 2.50 m from the edge. The shoot biomass was cut at the height of the cotyledon node. Non-N_2_-fixing species shoots were collected from each block: *Cyperus haspan* (papyrus sedge), *Eleusine indica* (goosegrass), and *Eragrostis plana* (tough lovegrass). Shoot biomasses of both mung bean and the non-N_2_-fixing species were placed in a forced circulation oven at 60 °C until reaching a constant mass.

The shoot N content for the six treatments was measured using the Kjeldahl method, while the Dumas method was employed for five treatments, except for the nitrogen control. The N content derived from BNF activity was determined using the ^15^N natural abundance technique. The following equation is used to calculate the BNF estimate:

(1) $$\mathrm{Ndfa}\%\;=\;\frac{\delta^{15}N_{reference\;plant}-\delta^{15}N_{legume}}{\delta^{15}N_{reference\;plant}-B}\times 100$$

In Equation (1), δ^15^N_reference plant_ indicates the soil δ^15^N obtained through non-fixing plants; δ^15^N_legume_ represents the δ^15^N of the N_2_-fixing plant. The B value, −2.5, represents the isotopic discrimination of ^15^N, where the proportion of ^15^N of the fixing plant is grown dependent only on BNF [23].

Grain yield was measured at 71 DAE when approximately 99% of the pods had reached the harvest stage. A 1.0 m^2^ section was marked within each experimental plot, including the third and fourth rows. Shoots were cut at the cotyledon node height and placed on benches to reduce leaf moisture. Then, the pods were detached and threshed. The constant mass of 100 grains was obtained in a forced circulation oven at 60 °C and corrected to 13% moisture.

### 2.3. Statistical Analysis

Normality of residuals and homogeneity of variance were assessed using the Shapiro–Wilk and Bartlett tests, respectively (*p* > 0.05). The nodule number variable was log-transformed. Data were analyzed with ANOVA using the F-test, and the means were compared with the Tukey test at the 5% significance level in R (v2020) [24]. Raw data are presented at Supplementary Data.

## 3. Results and Discussion

### 3.1. Selection of Rhizobial Strains for Mung Bean Under Controlled Conditions

Plant roots inoculated with the BR 3302, BR 96, or BR 3301 strains under axenic conditions showed a significantly higher nodule number, as analyzed by log (nodule number + 1), than the other ten strains and the non-inoculated control (Table 2; Supplementary S3). Inoculation with the BR 3302 strain resulted in significant increases in mung bean nodule, root, and shoot dry mass compared to other treatments (Table 2). Inoculation with the BR 96 and BR 3301 strains resulted in significantly lower nodule and shoot dry masses than inoculation with the BR 3302 strain, but still performed better than the other treatments.

Plants inoculated with the BR 3302 strain showed approximately five times higher shoot dry mass and 2.5 times higher root dry mass compared to the non-inoculated control. Meanwhile, the BR 96 and BR 3301 strains resulted in about three times higher shoot dry mass than the non-inoculated control.

Results obtained under axenic conditions demonstrate the viability of associations between mung beans and various rhizobial species, suggesting that the plant shows low selectivity toward the microsymbiont [7,25,26,27]. Experiments conducted in pots filled with soil from Ethiopia confirmed that *Vigna* species, such as mung beans and cowpeas, can be nodulated by several *Bradyrhizobium* groups [8]. Despite the low specificity toward microsymbionts, BNF in mung bean nodules has been recognized as highly efficient, accounting for a significant portion of the crop’s nitrogen [23,28].

The isolation and characterization of 101 bacterial strains from nodules of two mung bean cultivars grown in different Brazilian soils revealed that the *Bradyrhizobium* genus is involved in nodulation [6]. Biological nitrogen fixation (BNF) efficiency was influenced by the strain’s phylogenetic group and soil type [6]. This specificity may be related to the *nif*, *nod*, and *ysc* genes of *Bradyrhizobium* strains, which are involved in BNF, including nodulation, nitrogen (N) fixation, and secretion systems [29]. More specific genes, such as NopE (*B. diazoefficiens*) and NopP2 (*B. elkanii*), can affect the success of symbiosis between the microorganism and specific mung bean cultivars [30,31].

In our study, three of the eight slow-growing *Bradyrhizobium* strains successfully nodulated mung beans. Meanwhile, none of the three fast-growing *Rhizobium* strains induced nodulation.

Mung bean inoculation with strains isolated from cowpea nodules characteristic of the Amazon region (BR 3301 and BR 3302) and from soybean (BR 96) showed the best results in terms of nodulation and biomass accumulation (Table 2).

Favero et al. (2022a) [32] observed similar results in a pot experiment using soil, comparing mung bean inoculation with eight commercial inoculant strains (used for cowpea and soybean) and 13 *Bradyrhizobium* isolates from mung bean nodules. Among the tested strains, BR 3302, BR 3301, BR 96, and BR 3267 nodulated and promoted mung bean growth.

Similarly, the CB 1015 strain recommended for cowpea also effectively nodulates mung beans [23]. In addition to nodulating mung beans, the BR 96 strain can also nodulate cowpea, as observed by [33] under controlled conditions. The authors found that the BR 96 strain exhibited nodule efficiency, shoot dry matter, and total N levels comparable to those of the BR 3262 and BR 3267 strains, which are known to be efficient for cowpea.

Besides the mung bean’s ability to form significant nodulation with the BR 96 strain, the species can also nodulate with Type I *Bradyrhizobium* strains of the peanut group and some Type II strains [26]. The authors suggest that mung beans belong to the peanut cross-nodulation group.

Our results show that two *Bradyrhizobium* strains recommended for cowpea inoculation (BR 3267 and BR 3262) and one for soybean (BR 29) induced only a few nodules on mung bean roots, resulting in no significant increase in shoot biomass (Table 2).

### 3.2. Field Evaluation of Bradyrhizobium Strains as Inoculants for Mung Bean

The top nodulating strains from the previous experiment under axenic conditions (BR 3302, BR 3301, BR 3267, and BR 96) were selected as inoculants for a field trial. There were no significant differences in nodule numbers in response to seed inoculation with these strains (Table 3). Both inoculated and non-inoculated control plants had approximately 45 nodules per plant. The number of nodules produced by the indigenous community (non-inoculated control) in the present experiment was 4 and 10 times higher than those observed under field conditions reported by Hakim et al. (2020) and Bhuiyan and Mian (2007) [34,35], respectively, suggesting the presence of a high concentration of nodulating bacteria in the experimental area. According to Santos et al. (2020) [36], in a trial evaluating the growth of mung bean as a function of different nitrogen doses, a better response was observed at the dose of 240 kg N ha^−1^. Based on this result, we adopted a dose of 240 kg N ha^−1^ in the nitrogen control. It was observed that nodulation was reduced to around 25 nodules per plant in the nitrogen control.

Treatments inoculated with BR 96 and BR 3302 strains showed nodule dry masses more than 200% greater than the nitrogen control; however, there were no significant differences with BR 3267 and BR 3301 strains or the non-inoculated control. Data indicate the suppressive effect of N-fertilizer on mung bean nodulation (Table 3; Supplementary S4).

Since there has been no recent history of legume cultivation in this area, the abundant nodulation in non-inoculated plants was likely caused by an indigenous rhizobial population capable of nodulating mung beans (Table 3). It has been reported that mung beans tend to form nodules with various species of the *Bradyrhizobium* and *Ensifer* genera, and to a lesser extent with species of the *Mesorhizobium* and *Rhizobium* genera. Additionally, non-rhizobial endophytic species from *Proteobacteria, Actinobacteria*, *Bacteroidetes*, and *Firmicutes* have been found inside mung bean nodules [25,37,38,39,40].

Root nodules from the nitrogen control plot were noticeably smaller than those from other treatments, with a dry mass of about 1.2 mg each, while the nodules from the inoculated treatments and the non-inoculated control had a dry mass of approximately 2.0 mg. Smaller nodules indicate lower efficiency because fewer cells are infected with nitrogen-fixing bacteria.

**Figure 1:**
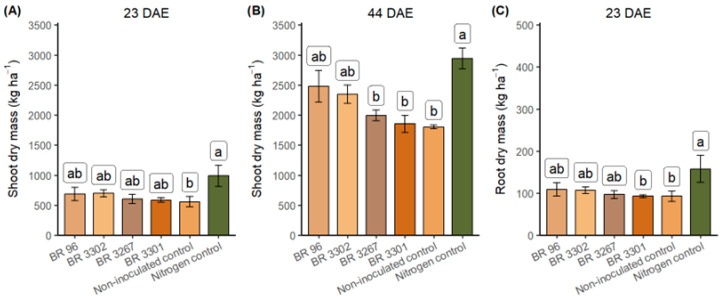
Shoot dry mass (SDM) (**A**) at 23 days after emergence (DAE) and (**B**) at 44 days after emergence; and root dry mass (RDM) (**C**) at 23 DAE of mung bean inoculated with *Bradyrhizobium* strains, alongside the non-inoculated and nitrogen (240 kg N ha^−1^) controls under field conditions. Shoot dry mass means (23 and 44 DAE) followed by different letters differ significantly from each other by the Tukey test at *p* < 0.05; and root dry mass means (23 DAE) followed by different letters vary considerably from each other by the Tukey test at *p* < 0.07.

Nitrogen application was significantly associated with higher shoot dry mass at 23 and 44 days after emergence (DAE), with increases of approximately 77% and 63%, respectively, compared to non-inoculated plants (Figure 1A,B; Supplementary S5). The shoot dry mass of plants inoculated with the BR 96 and BR 3302 strains did not significantly differ from that of the nitrogen control. Bhuiyan and Mian (2007) [35] evaluated five mung bean varieties in a field with good natural soil fertility. The authors found that inoculation with a *Bradyrhizobium* strain increased nodulation and shoot biomass by 55% and 27%, respectively, compared to non-inoculated plants. In contrast to the substantial nodulation observed in the roots of non-inoculated plants in our experiment, the results reported by Bhuiyan and Mian (2007) [35] suggest a low concentration of nodulating cells in the experimental area, despite its soil fertility.

Significant differences, identified by the Tukey test at a 7% probability level, were observed in root dry mass between the inoculated treatments and the non-inoculated and nitrogen controls, with values ranging from 90 to 160 kg ha^−1^ (Figure 1C; Supplementary S5). It should be noted that the fresh and dry root masses, as well as nodulation parameters, may have been underestimated, since both the clay texture and soil compaction make it difficult to remove the root system. These conditions typically result in only partial root and nodule recoveries [41], especially in clay soils like those in the experimental area. Depending on soil conditions, legumes such as mung beans develop deep roots tolerant of low humidity, which can contribute to greater data variability.

Nodulation in plants grown in soil-filled pots tends to produce more nodules. This is evident in the experiment described by Christopher et al. (2018) [23], where up to 85 nodules per root were observed in inoculated plants. Such high nodule numbers are rarely seen in field experiments, where the maximum was around 50 nodules per root. In a pot experiment, the number of nodules per gram of root was 345 [23]. In comparison, 86 nodules were obtained in the present field experiment, suggesting that evaluating nodulation under these conditions may be limited and should be interpreted carefully.

The nitrogen content of plants at 44 DAE, measured by the Kjeldahl digestion method, did not significantly differ among the inoculated treatments, the non-inoculated control, or the nitrogen control. However, the highest shoot N value was observed in plants receiving nitrogen, which was significantly different from treatments inoculated with BR 3267 and BR 3301 strains or from the non-inoculated control plants (Table 4; Supplementary S6). The total shoot N difference between the nitrogen control and the non-inoculated control was 45.7 kg ha^−1^. Plants inoculated with BR 96 and BR 3302 strains, which did not differ significantly from the nitrogen control, accumulated an average of 17.6 kg ha^−1^ of total N compared to the non-inoculated plants. Based on preliminary data, we estimated that the mung bean growth potential was reached at a dose of 240 kg N ha^−1^. Inoculation with the BR 96 and BR 3302 strains accounted for approximately 40% of the total N accumulated in the shoot, even with a high rate of nodulating bacteria in a clay soil with good natural fertility and organic matter.

Table 4 shows that the N content values obtained by the Dumas method did not reveal significant differences among the treatments, unlike those obtained by the Kjeldahl digestion method. The total N accumulated in the shoot indicated that inoculation with the BR 96 strain was 54% higher than with the BR 3301 strain. There were no significant differences between the other treatments. Mathu et al. (2012) [42] also did not observe significant differences in total N accumulation in mung bean shoots, whether promoted by inoculation with four commercial inoculants or by the non-inoculated control, in 10 soil samples from Kenya with a history of legume cultivation. Conversely, without prior legume cultivation, three mung bean genotypes inoculated with three *Bradyrhizobium* strains resulted in greater nodulation and higher N accumulation in plant biomass than non-inoculated plants [23]. The BR 96 strain also resulted in total N accumulation in cowpea shoots, similar to the BR 3267 and BR 3262 strains recommended for this crop [33].

The BR 3301 strain inoculation resulted in significantly higher nitrogen content derived from BNF, approximately twice that of the BR 3302 strain. No significant differences were observed among the other treatments or the non-inoculated control. The nitrogen levels in the air, measured after inoculation, ranged from 35.7% to 66.1%. In contrast, the indigenous soil population responsible for root nodulation in the non-inoculated control plants accounted for about 45% of the shoot N derived from the air. In experiments conducted in mung bean production areas, Herridge et al. (2005) [43] demonstrated that inoculation with *Bradyrhizobium* strains contributed to a 9% increase in air N_2_ content in the shoot compared to non-inoculated plants.

In a previous study by Mathu et al. (2012) [42], the N values obtained from the air ranged from 30% to 95% across soils with different textures: clay loam, sandy clay loam, sandy loam, and sand. The authors concluded that the highest N content was associated with sand. In our experiment, conducted in clay soil, a 45% N content derived from the air was observed in non-inoculated plants, which is close to the values reported by Mathu et al. (2012) [42] for plants grown in soil with a similar texture, such as clay loam. These authors also determined that the N levels derived from air in cowpea shoot dry mass ranged from 80 to 95% across textural classes, suggesting that *V. unguiculata* showed more uniform BNF activity than mung beans.

When evaluating shoot N derived from air in plants inoculated with the BR 96 strain, we observed increases of approximately 90% and 68%, respectively, compared to non-inoculated control plants and those inoculated with the BR 3302 strain. A similar trend was noted in the study by Christopher et al. (2018) [23], where the N fixed in the shoots of the two mung bean cultivars that were more responsive to inoculation was about 4.5 times higher than in the non-inoculated control.

Conversely, inoculation with the BR 3302 strain increases soil-derived shoot N values, ranging from 29.3% to 63.1% compared to the non-inoculated control or strains other than BR 3302. Although its efficiency in BNF was limited, the BR 3302 strain significantly enhances the plant’s ability to extract N from the soil.

Diatta et al. (2020) [28] determined shoot N fixed from the air and extracted from the soil of five mung bean genotypes grown in pots with soil from two textural classes, all receiving a single inoculant. The authors found that shoot N from the soil accounted for about 75% of the total N, regardless of the textural class or plant genotype. In our experiment, shoot N derived from the soil in plants inoculated with the BR 3302 strain was approximately 64% of the total N, while the percentage of N from the soil was around 40% for the other strains, which showed higher BNF efficiency. These results suggest that the difference in soil N uptake relative to total N is not due to soil texture or plant genotype. Instead, the response depends on strain traits related to BNF activity or the presence of a plant growth-promoting mechanism, as appears to be the case with the BR 3302 strain.

The studies cited above [23,28,42] used pots filled with soil to determine the N content derived from the atmosphere, but it should be noted that, under these conditions, the soil volume explored by the roots may be limited. Field experiments offer a better understanding of the physiological aspects of the symbiosis established between nodulating bacteria and mung beans. To our knowledge, so far, there is only the current field trial that has evaluated the fixation rates of atmospheric N_2_ by the ^15^N method in mung beans conducted in the American continent.

**Figure 2:**
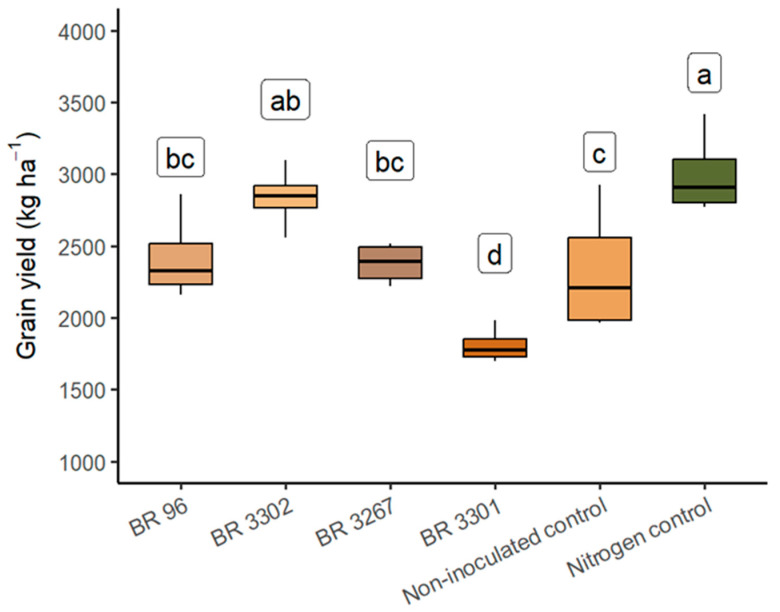
Grain yield of mung beans at 72 DAE, inoculated with *Bradyrhizobium* strains, non-inoculated, and nitrogen (240 kg N ha^−1^) controls under field conditions. Letters indicate mean values, and different letters show significant differences according to the Tukey test at *p* < 0.05.

The higher grain yield of mung bean resulted from inoculation with the BR 3302 strain, which reached about 2840 kg ha^−1^, 18% higher than the non-inoculated control and 36% higher than the BR 3301 strain inoculation. It did not differ significantly from the nitrogen control (3000 kg ha^−1^) (Figure 2; Supplementary S7). Inoculating mung bean seeds with the BR 3302 strain led to a grain yield increase of approximately 500 kg ha^−1^ compared to non-inoculated seeds. Furthermore, inoculation with the BR 96 and BR 3267 strains produced a grain yield roughly 25% higher than inoculation with the BR 3301 strain but did not significantly differ from the non-inoculated control.

Similar field results were reported by Bhuiyan and Mian (2007) [35], who demonstrated that inoculating mung beans with *Bradyrhizobium* strains led to an average increase in grain yield of 28% over two growing seasons. Hakim et al. (2020) [34] found a 25% increase in grain yield for plants co-inoculated with *Ensifer* sp. and *Acinetobacter* sp. compared to non-inoculated plants. The observed increases were independent of the productivity measured in the three experiments, which ranged from 0.6 to 2.8 Mg h^−1^. Alternatively, Herridge et al. (2005) [43] found that inoculating mung beans in field conditions resulted in smaller yield increases, approximately 6%, compared to non-inoculated plants. They concluded that seed inoculation might not be necessary under these conditions.

The BR 96 strain contributed the most to BNF until the start of the grain filling stage (44 DAE) (Table 4). However, grain yield in plants inoculated with BR 96 was about 15% lower than in those inoculated with BR 3302. We suggested that the results were due either to N_2_ fixation from the atmosphere or N uptake from the soil. Additionally, we calculated the BNF efficiency by subtracting the N fixed in each inoculated treatment from that in the non-inoculated plants, which showed that the indigenous population fixed 44.3% of atmospheric N. The BNF efficiency value reflects the strain’s behavior in relation to the specific edaphoclimatic conditions and the nodulating indigenous populations. From this calculation, we obtained 16.7 and 2.3 kg N ha^−1^ derived from BNF for BR 96 and BR 3302 strains, respectively, indicating that the BR 3302 strain had activity similar to the indigenous population. Despite the BR 3302 strain showing lower BNF efficiency compared to BR 96, the presence of plant growth-promoting mechanisms likely contributed to the higher grain yield promoted by the former.

Zilli et al. (2011) [33] calculated the nodular efficiency of the BR 96 strain in cowpea and observed similar values to the BR 3267 strain, which was selected for the crop. According to Neves and Hungria (1987) [44], the efficiency of the strain responsible for nodulation is reflected in increased grain yield. However, in this study, strain efficiency on mung bean grain yield did not necessarily follow this pattern, as seen with the BR 96 strain, which showed the best BNF activity but did not correspond to the one that led to the greatest grain yield (Figure 2).

**Figure 3:**
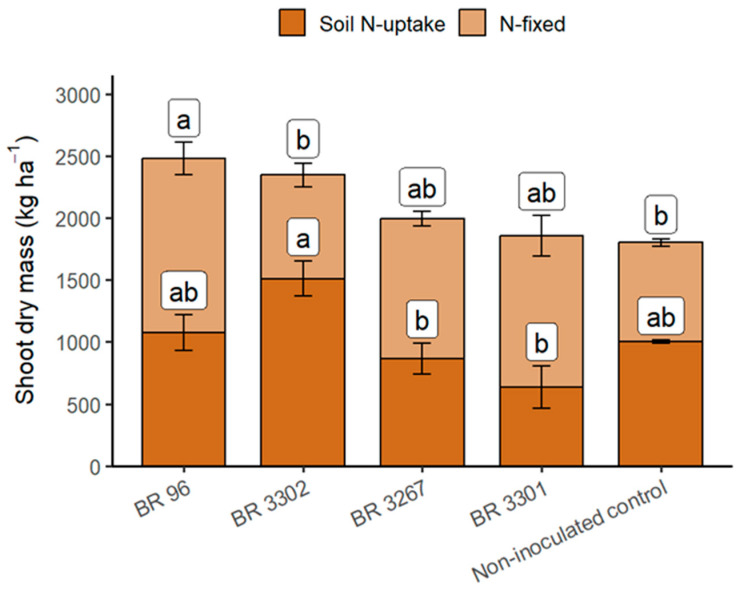
Distribution of dry shoot matter between nitrogen from BNF (N-fixed) and nitrogen from soil (Soil N-uptake) in mung beans at 44 DAE, inoculated with *Bradyrhizobium* strains and the non-inoculated control under field conditions. Means followed by different letters differ significantly based on the Tukey test at *p* < 0.05.

Figure 3 (Supplementary S8) displays the shoot biomass partition data related to N-fixed and soil N-uptake at 44 DAE. The highest shoot dry mass associated with N-fixed is from inoculation with the BR 96 strain, about 75% higher than non-inoculated plants, representing an increase of 612 kg ha^−1^. Meanwhile, the shoot proportion related to N-fixation in plants inoculated with the BR 3302 strain was similar to that of the non-inoculated control plants. However, BR 3302 inoculation resulted in approximately 50% higher shoot-associated soil N uptake than in non-inoculated plants, corresponding to an increase of 507 kg ha^−1^, which may have contributed to the increased grain yield (Figure 2).

Like other tropical legumes, mung beans mainly export N-fixed compounds through the xylem as ureides, although this compound decreases in the presence of soil nitrate [44]. Seed N accumulation is quite complex, involving compounds that arrive directly via the xylem and those that originate from protein turnover or mobilize leaf reserves, which are translocated by the phloem. According to Neves and Hungria (1987) [44], the increase in legume yield is linked to the ability to maintain BNF activity during the grain filling period, which can be achieved by selecting cultivars and/or strains. In this case, none of the inoculated mung beans seem to have maintained BNF during this period. Therefore, the N-extracting capacity of the BR 3302 strain may have significantly contributed to the results.

An estimated value of about 2.8 Mg N ha^−1^ is derived from soil organic matter, and a small part of this total is accessible to meet the crop’s low N needs. The results show that plants inoculated with the BR 3302 strain used the soil N reserve more efficiently.

Argawa and Tsigie (2017) [45] evaluated the BNF activity in common bean. They discovered that naturally high-fertility soils result in greater nodule production, which corresponds with higher grain yields compared to low-fertility soils. Therefore, it can be concluded that other legumes, including mung beans, can also benefit from soil fertility to improve BNF activity and soil nitrogen uptake.

**Figure 4:**
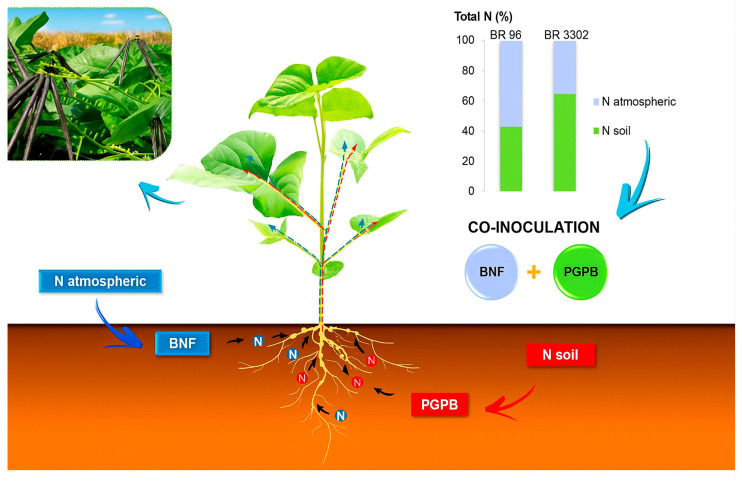
Typical diagram of biological nitrogen fixation (BNF) and plant growth-promoting bacteria (PGPB) activities in mung beans.

Based on the results from this experiment on predominantly clay soil with organic matter, we identified two main behaviors related to the microsymbiont: 1. A dominance of BNF activity, as observed with the BR 96 strain, which produced the highest shoot biomass until the grain filling stage; and 2. A dominance of plant growth promotion activity focused on soil N-uptake, linked to the BR 3302 strain, which increases grain yield. Given these points, it can be hypothesized that co-inoculation with both strains may enhance plant/microorganism efficiency regarding N availability (Figure 4; Supplementary S9).

In addition to the BNF contribution, Velázquez et al. (2019) [46], reported that bacteria from the rhizobium group also exhibited several plant growth-promoting activities, such as phosphate solubilization and the production of indole-3-acetic acid and ACC deaminase when studied in oilseeds, cereals, and vegetables. These authors show that PGPB activities may also be expressed in legumes, perhaps alongside atmospheric nitrogen fixation, and suggest that using rhizobia as a PGPB, considered safe for human, animal, and plant health, may be a good option for developing biofertilizer formulations.

Studies with cowpeas have shown that the BR 3302 strain’s benefits may extend beyond its ability to fix N_2_ from the air. The strain also demonstrates the capacity to perform additional functions, such as promoting plant growth by inducing phytohormone synthesis and conferring antibiotic resistance, which increases competitiveness against indigenous microbiota [47]. P and Ca solubilization traits are also features of the BR 3302 strain, potentially enhancing plant nutrition and improving plant performance across various edaphoclimatic conditions [48].

Therefore, further studies are needed to investigate the role of the BR 3302 strain in plant growth-promoting mechanisms and to evaluate the combined effect of BR 3302 and BR 96 strains on mung bean grain yield. New experiments involving inoculation with BR 3302 and BR 96 strains, as well as co-inoculation of both strains, could reveal a complementary relationship in the PGPR mechanisms between the two strains.

The results from the non-inoculated plants indicate that mung beans show low selectivity toward the microsymbiont, as they are well nodulated. These findings suggest that the native rhizobial population capable of nodulating mung beans is not very efficient at fixing atmospheric N_2_, implying that seed inoculation was beneficial under the experimental conditions.

## 4. Conclusions

This study demonstrates that *Bradyrhizobium* strains BR 3301, BR 3302, and BR 3267, recommended for cowpea, and BR 96 for soybean, can nodulate mung beans. Inoculation with the BR 96 strain (*B. elkanii*) increases shoot nitrogen content from atmospheric N_2_ fixation. Despite the high efficiency of the BR 96 strain, inoculation with BR 3302 (*B. viridifuturi*) resulted in an 18% increase in grain yield compared to the non-inoculated control, without significantly differing from the treatment that received 240 kg N ha^−1^. We suggest that the observed response may stem from a dual mechanism: biological nitrogen fixation and plant growth promotion.

## Figures and Tables

**Table 1:** List of rhizobial strains registered with MAPA *, other taxonomic designations, bacterial species, and references.

Leguminous Species from Which It Was Isolated	Bacterial Strains (SEMIA)	Other Designations	Bacterial Species **	Technical–Scientific Publications
Cowpea	SEMIA 6463	=BR 3301 or INPA 3-11B	*Bradyrhizobium amazonense*	de Souza Moreira et al. (2024) [11]
	SEMIA 6461	=BR 3302 and UFLA 3-84)	*Bradyrhizobium viridifuturi*	da Costa et al. (2019) [12]
	SEMIA 6462	=BR 3267	*Bradyrhizobium yuanmingense*	Simões-Araújo et al. (2016) [13]
	SEMIA 6464	=BR 3262	*Bradyrhizobium pachyrhizi*	Simões-Araújo et al. (2016) [13]
Soybean	SEMIA 5080	=BR 85	*Bradyrhizobium diazoefficiens*	Menna et al. (2009); Klepa et al. (2024) [14,15]
	SEMIA 5079	=BR 86	*Bradyrhizobium japonicum*	Menna et al. (2009); Klepa et al. (2024) [14,15]
	SEMIA 587	=BR 96	*Bradyrhizobium elkanii*	Menna et al. (2009); Klepa et al. (2024) [14,15]
	SEMIA 5019	=BR 29	*Bradyrhizobium elkanii*	Menna et al. (2009); Klepa et al. (2024) [14,15]
Common bean	SEMIA 4077	=BR 322	*Rhizobium tropici*	Dall’Agnol et al. (2013) [16]
	SEMIA 4088	=BR 534	*Rhizobium tropici*	Mostasso et al. (2002) [17]
	SEMIA 4080	=BR 520	*Rhizobium freirei*	Klepa et al. (2024) [15]

* MAPA, Brazilian Ministry of Agriculture and Livestock. ** Information on the 16S rRNA, *nodC*, and *nifH* genes from GenBank, including genome accession numbers, is presented as Supplementary S1.

**Table 2:** Mung bean inoculated with rhizobial strains registered at MAPA * for various leguminous plant species after 60 days of emergence under axenic conditions in a greenhouse: Nodule number (NN) analyzed from log (NN + 1), nodule dry mass (NDM), root dry mass (RDM), and shoot dry mass (SDM).

Rhizobial Strains	Leguminous Plant	NN	NDM	RDM	SDM
		Nodule Plant^−1^	mg Plant^−1^	g Plant^−1^	
BR 3302	cowpea	49 a **	138 a	0.294 a	0.930 a
BR 96	soybean	70 a	98 b	0.178 bc	0.582 b
BR 3301	cowpea	72 a	89 b	0.200 ab	0.590 b
BR 3262	cowpea	2 b	6 c	0.122 bc	0.222 c
BR 3267	cowpea	1 b	3 c	0.114 bc	0.212 c
BR 29	soybean	3 b	15 c	0.138 bc	0.264 c
BR 85	soybean	0 b	0 c	0.126 bc	0.182 c
BR 86	soybean	0 b	0 c	0.114 bc	0.190 c
BR 322	common bean	0 b	0 c	0.110 bc	0.174 c
BR 534	common bean	0 b	0 c	0.110 bc	0.204 c
BR 520	common bean	0 b	0 c	0.094 c	0.180 c
Non-inoculated control	-	0 b	0 c	0.112 bc	0.188 c
Treatment		*	*	*	*

* MAPA—Ministry of Agriculture and Livestock. ** Means followed by different letters in the same column differ significantly from each other by the Tukey test (* *p* < 0.05).

**Table 3:** Nodule number (NN) was calculated using log (NN + 1) and nodule dry mass (NDM) of mung bean plants at 23 days after emergence under field conditions.

Rhizobial Strains	NN	NDM
	Nodule Plant^−1^	mg Plant^−1^
BR 96	49.3	111.5 a *
BR 3302	43.1	96.0 a
BR 3267	44.1	92.8 ab
BR 3301	37.6	80.2 ab
Non-inoculated control	45.4	91.3 ab
Nitrogen control (240 kg N ha^−1^)	25.4	31.7 b
Treatment	ns	*

* Means followed by different letters within the same column differ significantly according to the Tukey test (ns: not significant, * *p* < 0.05).

**Table 4:** Shoot dry mass (SDM) and nitrogen (N) content measured by Kjeldahl and Dumas methods, total N (TN), N from biological nitrogen fixation (Ndfa), N sourced from the atmosphere (N-fixed), and N from soil (Soil N-uptake) in the shoot dry mass of mung beans at 44 days after emergence under field conditions.

Rhizobial Strains	SDM	N Content	TN	Ndfa	N-Fixed	Soil N-Uptake
	kg ha^−1^	%	kg ha^−1^	%	kg ha^−1^	
		Kjeldahl method				
BR 96	2479.3 ab	2.96	73.97 ab *	-	-	-
BR 3302	2347.3 ab	2.99	71.25 ab	-	-	-
BR 3267	1992.7 b	3.06	61.15 b	-	-	-
BR 3301	1854.2 b	2.71	50.47 b	-	-	-
Non-inoculated control	1802.9 b	3.04	54.97 b	-	-	-
Nitrogen control (240 kg N ha^−1^)	2941.0 a	3.40	100.66 a	-	-	-
Treatment	*	ns	*	nd	nd	nd
		Dumas method				
BR 96	2479.3	2.51	62.03 a *	57.12 ab	35.27 a	26.75 ab
BR 3302	2347.3	2.47	58.74 ab	35.75 b	20.88 b	37.86 a
BR 3267	1992.7	2.53	50.58 ab	57.02 ab	28.68 ab	21.90 b
BR 3301	1854.2	2.16	40.21 b	66.15 a	26.25 ab	13.96 b
Non-inoculated control	1802.9	2.32	41.93 ab	44.27 ab	18.54 b	23.38 ab
Treatment	ns	ns	*	*	*	*

* Means followed by different letters in the same column differ significantly according to the Tukey test (ns: not significant, * *p* < 0.05), and nd: not determined.

## Data Availability

The raw data presented in this study are available as Supplementary Materials.

## Supplementary Materials

The following supporting information can be downloaded at: https://www.mdpi.com/article/10.3390/plants14233695/s1, S1. Genomic identification of rhizobial strains used in the experiments, including GenBank accession numbers and coordinates for 16S rRNA and symbiotic genes (*nodC* and *nifH*); S2. How to prepare Norri’s nutrient Solution for Leonard’s jars; S3. Mung bean inoculated with rhizobial strains registered at MAPA for different legumionous plant species at 60 days after emergence under axenic conditions in a greenhouse: Nodule number (NN) analyzed from log (NN + 1), nodule dry mass (NDM), root dry mass (RDM) and shoot dry mass (SDM); S4. Nodule number (NN) analyzed from log NN and nodule dry mass (NDM) of mung bean plants at 23 days after emergence under field conditions; S5. Shoot dry mass (SDM) (a) in the 1st sampling day at 23 days after emergence and (b) in the 2nd sampling day at 44 days after emergence; and root dry mass (RDM) (c) in the 1st sampling day at 23 days after emergence of mung bean inoculated with Bradyrhizobium strains and the absolute (non-inoculated) and nitrogen (240 kg N ha^−1^) controls under field conditions; S6. Shoot dry mass (SDM) and N content by Kjeldahl and Dumas methods, total N (TN), N content derived from BNF (Ndfa), N derived from the atmosphere (N-fixed) and N derived from soil (Soil N-uptake) in the shoot dry mass of mung bean at 44 days after emergence under field conditions; S7. Grain yield of mung bean at 72 days after emergence inoculated with Bradyrhizobium strains and absolute (non-inoculated) and nitrogen (240 kg N ha^−1^) controls under field conditions; S8. Shoot dry matter partition corresponding to nitrogen derived from BNF (N-fixed) and nitrogen derived from soil (Soil N-uptake) in mung bean at 44 days after emergence, inoculated with Bradyrhizobium strains and absolute control (non-inoculated) under field conditions; S9. Representative scheme of biological nitrogen fixation (BNF) and plant growth-promoting bacteria (PGPB) activities in mung bean.
