# Supplementary material for: Harnessing Rhizobial Inoculation for Sustainable Nitrogen Management in Mung Bean (Vigna radiata L.)
Source: Plants (Basel). 2025 Dec 4;14(23):3695. doi: 10.3390/plants14233695 (PMC12694309; doi:10.3390/plants14233695)

S1. Genomic identification of rhizobial strains used in the experiments, including GenBank accession numbers and coordinates for 16S rRNA and symbiotic genes (*nodC* and *nifH*).

Host plant	Rhizobial Strains	Species	Gene	Access number	Genomic coordinates (bp)
**Cowpea**	BR 3301	***Bradyrhizobium amazonense*	16S rRNA	NZ_JASGSR010000002.1	80346-81834
	BR 3301	***Bradyrhizobium amazonense*	*nodC*	NZ_JASGSR010000090.1	20559-21890
	BR 3301	***Bradyrhizobium amazonense*	*nifH*	NZ_JASGSR010000068.1	17525-18409
	BR 3267	*Bradyrhizobium yuanmingense*	16S rRNA	NZ_LJYF01000029.1	889834-891322
	BR 3267	*Bradyrhizobium yuanmingense*	*nodC*	NZ_LJYF01000016.1	83928-85304
	BR 3267	*Bradyrhizobium yuanmingense*	*nifH*	NZ_LJYF01000029.1	129025-129909
	BR 3302	****Bradyrhizobium viridifuturi*	16S rRNA	NZ_NSJY01000003.1	272430-273918
	BR 3302	****Bradyrhizobium viridifuturi*	*nodC*	NZ_NSJY01000011.1	103184-104542
	BR 3302	****Bradyrhizobium viridifuturi*	*nifH*	NZ_NSJY01000020.1	24049-24933
	BR 3262	*Bradyrhizobium pachyrhizi*	16S rRNA	NZ_LJYE01000061.1	88496-89984
	BR 3262	*Bradyrhizobium pachyrhizi*	*nodC*	NZ_LJYE01000116.1	67725-69095
	BR 3262	*Bradyrhizobium pachyrhizi*	*nifH*	NZ_LJYE01000081.1	29175-30059
**Soybean**	BR 85	*Bradyrhizobium diazoefficiens*	16S rRNA	NZ_CP139636.1	8254299-8255787
	BR 85	*Bradyrhizobium diazoefficiens*	*nodC*	NZ_CP139636.1	7580260-7581717
	BR 85	*Bradyrhizobium diazoefficiens*	*nifH*	NZ_CP139636.1	7858563-7859447
	BR 86	*Bradyrhizobium japonicum*	16S rRNA	NZ_CP007569.1	3349549-3351037
	BR 86	*Bradyrhizobium japonicum*	*nodC*	NZ_CP007569.1	272016-273473
	BR 86	*Bradyrhizobium japonicum*	*nifH*	NZ_CP007569.1	9524975-9525859
	BR 96	*Bradyrhizobium elkanii*	16S rRNA	NZ_SWAO01000030.1	5708-7196
	BR 96	*Bradyrhizobium elkanii*	*nodC*	NZ_SWAO01000134.1	18136-19509
	BR 96	*Bradyrhizobium elkanii*	*nifH*	NZ_SWAO01000082.1	28844-29728
	BR 29	*Bradyrhizobium elkanii*	16S rRNA	NZ_SWAN01000018.1	128889-130377
	BR 29	*Bradyrhizobium elkanii*	*nodC*	NZ_SWAN01000027.1	18212-19585
	BR 29	*Bradyrhizobium elkanii*	*nifH*	NZ_SWAN01000056.1	28200-29084
**Common bean**	BR 322	*Rhizobium tropici**	16S rRNA	NC_020059.1	69017-70497
	BR 322	*Rhizobium tropici**	*nodC*	NC_020061.1	236925-238283
	BR 322	*Rhizobium tropici**	*nifH*	NC_020061.1	285319-286212
	BR 534	*Rhizobium tropici*	16S rRNA	Not found	Not found
	BR 534	*Rhizobium tropici*	*nodC*	Not found	Not found
	BR 534	*Rhizobium tropici*	*nifH*	Not found	Not found
	BR 520	*Rhizobium freirei*	16S rRNA	Not found	Not found
	BR 520	*Rhizobium freirei*	*nodC*	Not found	Not found
	BR 520	*Rhizobium freirei*	*nifH*	Not found	Not found

*The genome of *Rhizobium tropici* BR 322 (CIAT 899) contains multiple copies of the 16S rRNA gene located on distinct contigs; the coordinates provided correspond to the first copy (rrnA operon).

**Listed in GenBank as *Bradyrhizobium* sp. INPA03-11B; reclassified as *Bradyrhizobium amazonense* by de Souza Moreira et al. (2024).
***Listed in GenBank as *Bradyrhizobium* sp. UFLA03-84; reclassified as *Bradyrhizobium viridifuturi* by da Costa et al. (2019).

S2. How to prepare Norri's nutrient Solution for Leonard's jars.

(1)	KCl	5.96 g	For 40 liters of distilled, deionized, and sterilized water.
(2)	K_2HPO_4	2.00 g	
(3)	KH_2PO_4	4.00 g	
(4)	$CaSO_4.2H_2O$	13.76 g	
(5)	$MgSO_4.7H_2O$	19.72 g	
(6)	$CuSO_4.5H_2O$	0.15 g	Fill to 1,000 mL with distilled water.
	$ZnSO_4.7H_2O$	0.44 g	
	$MnSO_4.2H_2O$	0.40 g	
	$(NH_4)_6Mo_7O_{24}.4H_2O$	0.02 g	
	H_3BO_3	1.43 g	
(7)	$FeSO_4.7H_2O$	5.00 g	Fill to 1,000 mL with distilled water.
	Citric Acid	5.00 g	

For 40 liters of water, add: (1), (2), (3), and (5) and dissolve.
Pipette 20 mL of solution (6) and 40 mL of solution (7) and add (4) when using the solution.

S3. Mung bean inoculated with rhizobial strains registered at MAPA for different legumionous plant species at 60 days after emergence under axenic conditions in a greenhouse: Nodule number (NN) analyzed from log (NN+1), nodule dry mass (NDM), root dry mass (RDM) and shoot dry mass (SDM).

Treatment	Block	NN (nodule/plant)	logNN+1 (nodule/plant)	NDM (mg/plant)	RDM (g/plant)	SDM (g/plant)
BR3302	1	35	1.556	131.00	0.19	0.99
BR3302	2	76	1.886	145.00	0.23	0.88
BR3302	3	36	1.568	135.00	0.33	0.93
BR3302	4	43	1.643	155.00	0.34	1.01
BR3302	5	54	1.740	124.00	0.38	0.84
BR96	1	60	1.785	102.00	0.17	0.67
BR96	2	73	1.869	150.00	0.28	0.84
BR96	3	101	2.009	83.00	0.16	0.45
BR96	4	68	1.839	103.00	0.18	0.69
BR96	5	48	1.690	53.00	0.10	0.26
BR3301	1	60	1.785	116.00	0.28	0.74
BR3301	2	116	2.068	87.00	0.15	0.67
BR3301	3	61	1.792	57.00	0.22	0.34
BR3301	4	78	1.898	106.00	0.18	0.67
BR3301	5	44	1.653	81.00	0.17	0.53
BR3262	1	0	0.000	0.00	0.10	0.21
BR3262	2	6	0.845	20.00	0.10	0.21
BR3262	3	2	0.477	12.00	0.14	0.28
BR3262	4	0	0.000	0.00	0.11	0.22
BR3262	5	0	0.000	0.00	0.16	0.19
BR3267	1	0	0.000	0.00	0.08	0.19
BR3267	2	0	0.000	0.00	0.12	0.20
BR3267	3	4	0.699	18.00	0.15	0.25
BR3267	4	0	0.000	0.00	0.12	0.24
BR3267	5	0	0.000	0.00	0.10	0.18
BR29	1	6	0.845	0.00	0.10	0.15
BR29	2	0	0.000	0.00	0.11	0.18

BR29	3	7	0.903	77.00	0.24	0.63
BR29	4	0	0.000	0.00	0.10	0.18
BR29	5	0	0.000	0.00	0.14	0.18
BR85	1	0	0.000	0.00	0.14	0.22
BR85	2	0	0.000	0.00	0.14	0.17
BR85	3	0	0.000	0.00	0.13	0.17
BR85	4	0	0.000	0.00	0.08	0.16
BR85	5	0	0.000	0.00	0.14	0.19
BR86	1	0	0.000	0.00	0.10	0.19
BR86	2	0	0.000	0.00	0.12	0.22
BR86	3	0	0.000	0.00	0.11	0.21
BR86	4	0	0.000	0.00	0.12	0.14
BR86	5	0	0.000	0.00	0.12	0.19
BR322	1	0	0.000	0.00	0.10	0.18
BR322	2	0	0.000	0.00	0.11	0.12
BR322	3	0	0.000	0.00	0.16	0.19
BR322	4	0	0.000	0.00	0.09	0.20
BR322	5	0	0.000	0.00	0.09	0.18
BR534	1	0	0.000	0.00	0.10	0.19
BR534	2	0	0.000	0.00	0.12	0.30
BR534	3	0	0.000	0.00	0.12	0.18
BR534	4	0	0.000	0.00	0.15	0.18
BR534	5	0	0.000	0.00	0.06	0.17
BR520	1	0	0.000	0.00	0.08	0.16
BR520	2	0	0.000	0.00	0.08	0.20
BR520	3	0	0.000	0.00	0.09	0.14
BR520	4	0	0.000	0.00	0.14	0.27
BR520	5	0	0.000	0.00	0.08	0.13
Non-inoculated control	1	0	0.000	0.00	0.06	0.13
Non-inoculated control	2	0	0.000	0.00	0.09	0.18
Non-inoculated control	3	0	0.000	0.00	0.14	0.22
Non-inoculated control	4	0	0.000	0.00	0.17	0.21
Non-inoculated control	5	0	0.000	0.00	0.10	0.20

S4. Nodule number (NN) analyzed from log NN and nodule dry mass (NDM) of mung bean plants at 23 days after emergence under field conditions.

Treatment	Block	NN (nodule/plant)	logNN (nodule/plant)	NDM (mg/plant)
BR96	1	59.50	1.77	102.67
BR96	2	50.90	1.71	130.80
BR96	3	43.00	1.63	111.83
BR96	4	43.83	1.64	100.67
BR3302	1	31.17	1.49	52.50
BR3302	2	43.29	1.64	87.14
BR3302	3	67.25	1.83	165.75
BR3302	4	30.63	1.49	78.63
BR3267	1	71.38	1.85	106.63
BR3267	2	35.80	1.55	108.60
BR3267	3	45.63	1.66	100.50
BR3267	4	23.43	1.37	55.57
BR3301	1	50.67	1.70	96.83
BR3301	2	45.63	1.66	83.00
BR3301	3	30.83	1.49	82.33
BR3301	4	23.29	1.37	58.57
Non-inoculated control	1	26.63	1.43	51.00
Non-inoculated control	2	60.88	1.78	137.63
Non-inoculated control	3	37.63	1.58	77.50
Non-inoculated control	4	56.33	1.75	99.00
Nitrogen control	1	16.33	1.21	24.17
Nitrogen control	2	52.86	1.72	65.43
Nitrogen control	3	15.67	1.19	13.67
Nitrogen control	4	16.83	1.23	23.50

S5. Shoot dry mass (SDM) (**a**) in the 1st sampling day at 23 days after emergence and (**b**) in the 2nd sampling day at 44 days after emergence; and root dry mass (RDM) (**c**) in the 1st sampling day at 23 days after emergence of mung bean inoculated with *Bradyrhizobium* strains and the absolute (non-inoculated) and nitrogen (240 kg N ha^{-1}) controls under field conditions.

Treatment	Block	SDM (kg/ha)	SDM (kg/ha)	RDM (kg/ha)
		**Fig. 1a**	**Fig. 1b**	**Fig. 1c**
BR96	1	947.68	3042.18	146.12
BR96	2	573.34	2422.61	91.01
BR96	3	437.89	2668.66	75.61
BR96	4	783.47	1783.56	122.92
BR3302	1	648.95	2704.84	102.05
BR3302	2	604.35	1980.06	94.63
BR3302	3	651.27	2275.65	101.93
BR3302	4	878.45	2428.74	129.42
BR3267	1	536.46	2004.56	98.11
BR3267	2	473.70	2242.25	85.72
BR3267	3	587.95	1874.30	80.71
BR3267	4	812.30	1849.80	122.86
BR3301	1	607.20	1672.78	96.95
BR3301	2	498.54	1636.60	89.06
BR3301	3	674.93	1854.00	100.66
BR3301	4	550.28	2253.39	83.10
Non-inoculated control	1	534.03	1741.25	94.63
Non-inoculated control	2	568.12	1898.79	93.59
Non-inoculated control	3	353.81	1768.53	61.58
Non-inoculated control	4	767.24	1803.00	120.14
Nitrogen control	1	1339.65	2955.34	217.09
Nitrogen control	2	1186.84	2542.30	203.17
Nitrogen control	3	558.96	3374.51	81.18
Nitrogen control	4	860.94	2891.88	128.49

S6. Shoot dry mass (SDM) and N content by Kjeldahl and Dumas methods, total N (TN), N content derived from BNF (Ndfa), N derived from the atmosphere (N-fixed) and N derived from soil (Soil N-uptake) in the shoot dry mass of mung bean at 44 days after emergence under field conditions.

Treatment	Block	SDM (kg/ha)	N content (%)	TN (kg/ha)
		**Kjeldahl method**		
BR96	1	3042.18	3.10	94.28
BR96	2	2422.61	2.55	61.85
BR96	3	2668.66	3.26	87.08
BR96	4	1783.56	2.95	52.69
BR3302	1	2704.84	3.48	93.99
BR3302	2	1980.06	2.53	50.08
BR3302	3	2275.65	2.89	65.68
BR3302	4	2428.74	3.10	75.27
BR3267	1	2004.56	3.16	63.36
BR3267	2	2242.25	3.17	71.10
BR3267	3	1874.30	2.91	54.45
BR3267	4	1849.80	3.01	55.72
BR3301	1	1672.78	3.17	53.04
BR3301	2	1636.60	2.29	37.45
BR3301	3	1854.00	2.66	49.30
BR3301	4	2253.39	2.76	62.10
Non-inoculated control	1	1741.25	2.70	47.07
Non-inoculated control	2	1898.79	3.23	61.41
Non-inoculated control	3	1768.53	2.74	48.40
Non-inoculated control	4	1803.00	3.49	63.00
Nitrogen control	1	2955.34	3.91	115.41
Nitrogen control	2	2542.30	2.68	68.21
Nitrogen control	3	3374.51	3.28	110.75
Nitrogen control	4	2891.88	3.75	108.30

Treatment	Block	SDM (kg/ha)	N content (%)	TN (kg/ha)	Ndfa (%)	N fixed (kg/ha)	Soil N-uptake (kg/ha)
	**Dumas method**						
BR96	1	3042.18	2.54	77.29	57.12	44.15	33.14
BR96	2	2422.61	2.27	55.10	52.51	28.93	26.17
BR96	3	2668.66	2.51	66.94	55.25	36.98	29.96
BR96	4	1783.56	2.74	48.80	63.62	31.05	17.76
BR3302	1	2704.84	2.73	73.80	28.93	21.35	52.44
BR3302	2	1980.06	2.05	40.62	32.93	13.38	27.24
BR3302	3	2275.65	2.70	61.37	35.75	21.94	39.43
BR3302	4	2428.74	2.44	59.20	45.39	26.87	32.33
BR3267	1	2004.56	2.89	58.03	57.00	33.08	24.95
BR3267	2	2242.25	2.47	55.33	47.17	26.10	29.23
BR3267	3	1874.30	2.53	47.48	68.95	32.74	14.74
BR3267	4	1849.80	2.24	41.49	54.99	22.81	18.68
BR3301	1	1672.78	2.45	40.93	61.39	25.13	15.80
BR3301	2	1636.60	1.88	30.83	89.11	27.48	3.36
BR3301	3	1854.00	2.02	37.38	46.16	17.25	20.12
BR3301	4	2253.39	2.29	51.70	67.98	35.15	16.55
Non-inoculated control	1	1741.25	2.36	41.15	43.60	17.94	23.21
Non-inoculated control	2	1898.79	2.21	42.03	46.60	19.58	22.44
Non-inoculated control	3	1768.53	2.13	37.69	44.28	16.69	21.00
Non-inoculated control	4	1803.00	2.60	46.86	42.63	19.97	26.88

S7. Grain yield of mung bean at 72 days after emergence inoculated with Bradyrhizobium strains and absolute (non-inoculated) and nitrogen (240 kg N ha-1) controls under field conditions.

Treatment	Block	Grain yield (kg/ha)
BR96	1	2861.0
BR96	2	2401.6
BR96	3	2161.8
BR96	4	2255.0
BR3302	1	3099.6
BR3302	2	2858.9
BR3302	3	2837.0
BR3302	4	2555.3
BR3267	1	2485.7
BR3267	2	2517.5
BR3267	3	2218.7
BR3267	4	2291.5
BR3301	1	1695.7
BR3301	2	1981.0
BR3301	3	1805.0
BR3301	4	1740.9
Non-inoculated control	1	2926.4
Non-inoculated control	2	2430.9
Non-inoculated control	3	1988.1
Non-inoculated control	4	1962.1
Nitrogen control	1	3417.3
Nitrogen control	2	2812.7
Nitrogen control	3	2770.6
Nitrogen control	4	3000.0

S8. Shoot dry matter partition corresponding to nitrogen derived from BNF (N-fixed) and nitrogen derived from soil (Soil N-uptake) in mung bean at 44 days after emergence, inoculated with *Bradyrhizobium* strains and absolute control (non-inoculated) under field conditions.

Treatment	Block	SDM (kg/ha)	N content (%)	TN (kg/ha)	Ndfa (%)	N fixed (kg/ha)	Soil N-uptake (kg/ha)	SDM (N-fixed) (kg/ha)	SDM (Soil N-uptake) (kg/ha)
BR96	1	3042.18	2.54	77.29	57.12	44.15	33.14	1737.83	1304.35
BR96	2	2422.61	2.27	55.10	52.51	28.93	26.17	1272.10	1150.51
BR96	3	2668.66	2.51	66.94	55.25	36.98	29.96	1474.38	1194.28
BR96	4	1783.56	2.74	48.80	63.62	31.05	17.76	1134.63	648.93
BR3302	1	2704.84	2.73	73.80	28.93	21.35	52.44	782.61	1922.23
BR3302	2	1980.06	2.05	40.62	32.93	13.38	27.24	652.05	1328.01
BR3302	3	2275.65	2.70	61.37	35.75	21.94	39.43	813.55	1462.11
BR3302	4	2428.74	2.44	59.20	45.39	26.87	32.33	1102.48	1326.26
BR3267	1	2004.56	2.89	58.03	57.00	33.08	24.95	1142.60	861.96
BR3267	2	2242.25	2.47	55.33	47.17	26.10	29.23	1057.68	1184.57
BR3267	3	1874.30	2.53	47.48	68.95	32.74	14.74	1292.29	582.00
BR3267	4	1849.80	2.24	41.49	54.99	22.81	18.68	1017.12	832.68
BR3301	1	1672.78	2.45	40.93	61.39	25.13	15.80	1026.91	645.87
BR3301	2	1636.60	1.88	30.83	89.11	27.48	3.36	1458.34	178.26
BR3301	3	1854.00	2.02	37.38	46.16	17.25	20.12	855.82	998.18
BR3301	4	2253.39	2.29	51.70	67.98	35.15	16.55	1531.85	721.53
Non-inoculated control	1	1741.25	2.36	41.15	43.60	17.94	23.21	759.22	982.03
Non-inoculated control	2	1898.79	2.21	42.03	46.60	19.58	22.44	884.77	1014.02
Non-inoculated control	3	1768.53	2.13	37.69	44.28	16.69	21.00	783.02	985.51
Non-inoculated control	4	1803.00	2.60	46.86	42.63	19.97	26.88	768.56	1034.44

S9. Representative scheme of biological nitrogen fixation (BNF) and plant growth-promoting bacteria (PGPB) activities in mung bean.

	N soil	N FBN
BR 96	26.75	35.27
BR 3302	37.86	20.88

Extracted from the data in Figure 3.

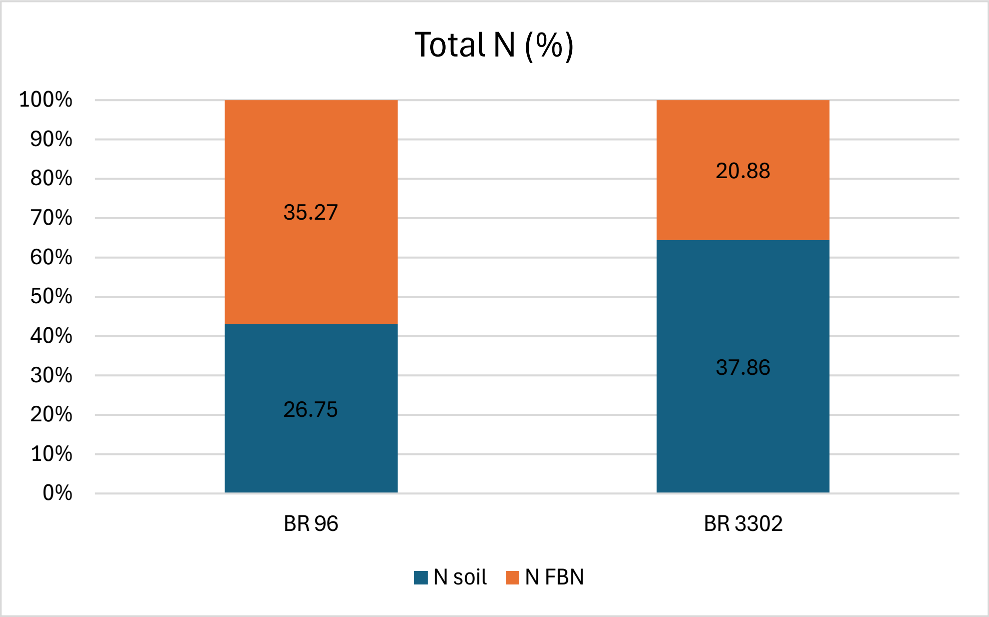

Supplement: Supplementary file 1 [file plants-14-03695-s001.zip › plants-3949664-supplementary.pdf]
